# Deep learning segmentation and quantification method for assessing epicardial adipose tissue in CT calcium score scans

**DOI:** 10.1038/s41598-022-06351-z

**Published:** 2022-02-10

**Authors:** Ammar Hoori, Tao Hu, Juhwan Lee, Sadeer Al-Kindi, Sanjay Rajagopalan, David L. Wilson

**Affiliations:** 1 Department of Biomedical Engineering, Case Western Reserve University, Cleveland, OH 44106 USA; 2 Department of Cardiology, University Hospitals Cleveland Medical Center, Cleveland, OH 44106 USA; 3 Department of Radiology, Case Western Reserve University, Cleveland, OH 44106 USA

**Keywords:** Imaging, Software, Cardiovascular models

## Abstract

Epicardial adipose tissue volume (EAT) has been linked to coronary artery disease and the risk of major adverse cardiac events. As manual quantification of EAT is time-consuming, requires specialized training, and is prone to human error, we developed a deep learning method (DeepFat) for the automatic assessment of EAT on non-contrast low-dose CT calcium score images. Our DeepFat intuitively segmented the tissue enclosed by the pericardial sac on axial slices, using two preprocessing steps. First, we applied a *HU-attention-window* with a window/level 350/40-HU to draw attention to the sac and reduce numerical errors. Second, we applied a novel *look ahead slab-of-slices with bisection* (“*bisect*”) in which we split the heart into halves and sequenced the lower half from bottom-to-middle and the upper half from top-to-middle, thereby presenting an always increasing curvature of the sac to the network. EAT volume was obtained by thresholding voxels within the sac in the fat window (− 190/− 30-HU). Compared to manual segmentation, our algorithm gave excellent results with volume Dice = 88.52% ± 3.3, slice Dice = 87.70% ± 7.5, EAT error = 0.5% ± 8.1, and R = 98.52% (p < 0.001). HU-attention-window and bisect improved Dice volume scores by 0.49% and 3.2% absolute, respectively. Variability between analysts was comparable to variability with DeepFat. Results compared favorably to those of previous publications.

## Introduction

Epicardial and paracardial fat have been linked to increased risk of cardiovascular disease and diabetes. Epicardial adipose tissue (EAT) is a visceral fat deposit distributed between the pericardium and the heart. Several clinical studies have shown a significant association between EAT volume and abdominal visceral adiposity^1,2^. A 2018 meta-analysis using CT images with > 41,000 participants over 70 studies showed an association between EAT volume and adverse cardiovascular risk^3^. Importantly, studies have shown a lack of (or weak) association between EAT and another widely used marker of risk, coronary artery calcium scoring^4,5^, suggesting that EAT volume may have additive value in risk stratification^6^. Emerging literature also suggests that EAT attenuation carries prognostic information^7–9^. Further, recent studies have shown that EAT is modifiable via pharmacologic treatment and may be a therapeutic target^10–12^. Manual EAT segmentation on non-contrast-enhanced CT images, however, is a time-consuming task, requires skilled expertise, and is prone to inter- and intra-observer variability^13^. For typical manual analysis, EAT is segmented by first delineating the pericardial sac and then thresholding voxels within the sac using the fat window (− 190 HU to − 30 HU). Yet, the thin layer of pericardium tissue can be difficult to distinguish in cardiac CT scans, with low contrast from surrounding tissues and blood^14^.

Recent publications have described the use of machine and deep learning approaches to segment EAT on non-contrast CT calcium score images^13^, contrast CT images^14^, and high-resolution CT angiography (CTA) images^15,16^. Some studies assessed both epicardial and paracardial (external to the pericardium) fat depots^17^, while others distinguished between epicardial and paracardial fat^13–15^. Some authors have used methods without learning, including a recent method by De Albuquerque et al.^16^, which used the floor of the log clustering algorithm and a set of morphological operations. Deep learning is popular using 2D slice^13^ and 3D patch^15^ data. Zhao et al*.*^14^ demonstrated a 2D Dense U-Net for automatically segmenting epicardium in 14 contrast-enhanced CTA images, where the increased contrast facilitates segmentation. He et al.^18^, proposed a 3D deep attention U-Net for segmenting the EAT in 40 CTA images. Their method achieved a Dice score of 85%. By extending their cohort to 200 CTA images^15^, the 3D deep attention U-Net approach reached an improved Dice score of 88.7%. For non-contrast gated, CT images, Zhang et al*.*^19^ applied a dual U-Net framework on 2D images slices over a small cohort (n = 20 image volumes).

With non-contrast CT images, Commandeur et al.^20^, proposed a fully automatic method that uses two Convolutional Neural Networks (CNNs) to segment EAT and thoracic adipose tissue (TAT). The first CNN detects the heart limits and performs segmentations while the second combines a statistical shape model to detect the pericardium. Our work is influenced by a subsequent paper from Commandeur et al.^5^, where they used a single deep learning approach in two tasks. First, they trained a deep network to segment the region within the pericardial sac. Second, they extracted features from the same network with machine learning to classify image slices containing the heart. The input to the semantic segmentation network consisted of a slab of three images slices, the slice of interest $(k)$, one prior $(k-1)$, and one post $(k+1)$. The output was a label image for the middle slice $(k)$. The use of three consecutive slices improved results significantly. However, when we applied this 3-slice approach, we found errors particularly associated with the top and bottom slices, leading us to develop an alternative approach.

Our goal was to perform an accurate, fully-automated EAT segmentation and quantification from CT calcium score images. CT calcium scoring is currently used to assess the cardiovascular health of patients, and large archives of thousands of CT calcium score images are available that enable population risk studies. However, CT calcium score images are challenging to analyze because the slices are thick (~ 2.5 mm), and no contrast agent is used to improve delineation of fat boundaries. CTA images reduce these challenges, but large CTA cohorts are less available, and CTA imaging is not used as a screening exam like CT calcium score imaging. Building on the work of Commandeur et al.^13^, we applied *HU-attention-window* with a window/level of 350/40 HU to emphasize appropriate CT numbers. Second, we applied a novel *look ahead slab-of-slices with bisection* (hereafter referred to as *bisect*) in which we split the heart into halves and sequenced the lower half from bottom-to-middle and the upper half from top-to-middle, thereby presenting an increased curvature of the sac to the network. We then used a 3-slice slab approach, with the image of interest at $(k)$ and other images at $(k+1)$, and $(k+2)$. In addition, we introduced a slice-based analysis of results for detailed quantifications that may be helpful for optimizing algorithms.

## Image analysis methods

### Manual labeling of image data

**Figure 1:**
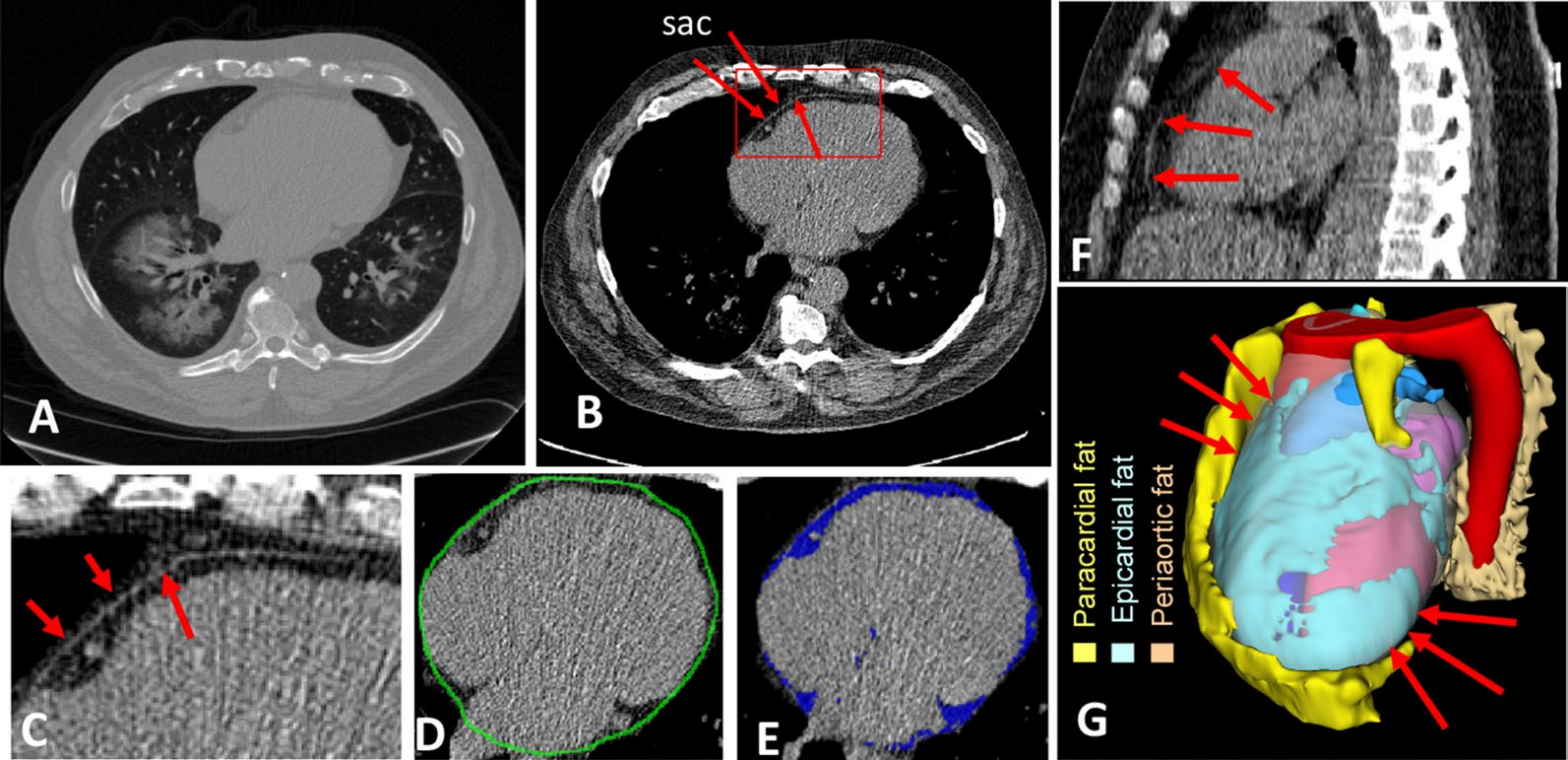
Manual segmentation of epicardial adipose tissue (EAT) on non-contrast CT images. Each 2D axial slice is displayed as in (**A**). The HU-attention-window with window/level of 350 HU/40 HU improved visualization of the pericardium (**B**). The pericardium (or pericardial sac) is marked with arrows in the inferior region (**B**) with the area bounded in red expanded in panel (**C**). In addition to axial views, we often rely on sagittal views to help identify the pericardium when the location is unclear (**F**). The expert analyst draws contours to distinguish the pericardium, shown in green in (**D**). Finally, EAT is identified as interior voxels thresholded within the fat window [− 190 HU, − 30 HU], as shown in blue in (**E**). A 3D demonstration of different fat tissues surrounding the heart is shown in (**G**), where EAT, shown in cyan, is enclosed by the sac, shown in transparent white, paracardial fat is above the sac, shown in yellow, while the periaortic fat, shown in light brown, is surrounding the aorta shown in red.

All scans in this study were obtained as part of clinical care. The Institutional Review Board of the University Hospitals waived consent for all studies utilizing anonymized CT scans. Method was carried out in accordance with relevant guidelines and regulations. Expert analysts segmented the CT scans using 3D-Slicer in a sequential slice-by-slice process (Fig. 1). The top and bottom of the heart were identified. A standard window/level (350 HU/40 HU) was applied to the entire CT volume to achieve good contrast of the pericardium (Fig. 1B). Analysts typically began the process in the middle of the heart. A closed region was manually drawn on every axial slice along the pericardium as in Fig. 1D. The anterior limit of the pericardium was determined by the appearance of the pericardium in both axial and sagittal views, as illustrated in Figs. 1C,F, respectively. If needed, axial slices above and below the current slice were examined to help determine the location of the pericardium. A median filter with a 3 × 3 × 3 mm kernel size was used to reduce noise. EAT fat was identified by thresholding in the standard fat range [− 190 HU, − 30 HU], and voxels within the pericardium were deemed EAT voxels, as shown in Fig. 1E. Manual segmentations were individually performed by three expert analysts for the 89 CT scan volumes. At the top and bottom regions of the heart, manual labeling became more difficult (as demonstrated in Fig. 1G and as will be shown with inter-reader variability results) and analysts used the sagittal view along with the axial view to enable precise labeling. Figure 1G was created using 3D slicer software (version 4.11)^21^.

### Algorithm for EAT segmentation

**Figure 2:**
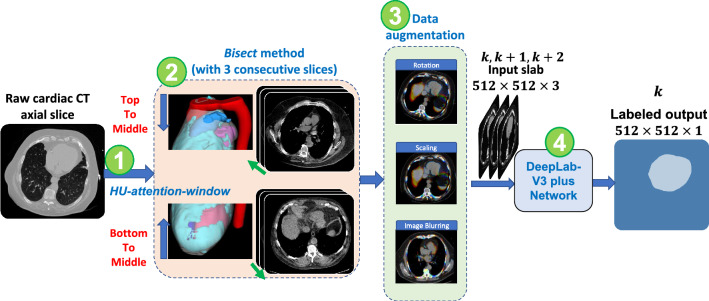
The full structure of the automated EAT segmentation training process and the preprocessing steps. A HU-attention-window/level of 40 HU/350 HU is shown in (1). A look ahead slab-of-slices with increasing size is presented to the network with the slice of interest and two up-coming slices on the increasing side, as in (2). We divide the heart slices into two halves where the lower half is sequenced from bottom-to-middle and the upper half sequenced top-to-middle, thereby keeping an increasing curvature of the sac and presenting similar images to the network in training (bisect method), as in (2). Different data augmentations enrich the deep learning with variations of cases, shown in (3). Finally, the DeepLab-v3 plus network is trained with each of the three sequenced patches with a single corresponding mask slice as in (4).

With experience from manual segmentation, we created preprocessing steps for our deep learning network (Fig. 2). For the *HU-attention-window*, we applied a 350 HU/40 HU window/level operation to increase the contrast of the pericardium and encourage the deep learning network to capture pericardium structural features. As processing within the network is done on 8-bit data, creating this truncation operation ensured that when data are mapped to the network, the pericardium contrast is not lost due to numerical rounding. We applied *look ahead slab-of-slices with bisection* (*bisect*) whereby we presented the network with the slice of interest and two upcoming slices on the side of increasing sac area. The cross-sectional area of the heart first enlarges and then narrows as one goes from the bottom of the heart to the top of the heart. As a result, we divided the heart slices into two equal halves where the lower half was sequenced from bottom-to-middle and the upper half top-to-middle, thereby keeping an increasing curvature of the sac and presenting similar images to the network in training and testing. We know from previous experience and from evidence in results that deep learning performs better when trained on a similar trend of images. Once data were arranged in this fashion, each $k$ labeled image slice of interest was concatenated with its consecutive two slices ($k+1$ and $k+2$) to generate a 512 × 512 × 3 input voxel slab for deep learning segmentation.

We segmented the region interior to the pericardial sac using deep learning semantic segmentation and used thresholding to determine EAT. We used DeepLab-v3-plus^22^ with transfer learning (i.e., the network was pre-trained on the ImageNet dataset), which uses Resnet-18 as a backbone. The deep network model is a CNN specifically designed for semantic segmentation tasks and is mainly composed of several important architectures: the backbone network, the Atrous convolution, the Atrous Spatial Pyramid Pooling (ASPP) network, and the decoder section, as shown in Fig. S1. Traditional deep CNNs tend to reduce the spatial resolution of the output feature map as the network goes deeper, and thus are not suitable for semantic segmentation tasks, which require detailed spatial information. In contrast to CNNs, the DeepLab-v3 plus applies Atrous convolution, which can adjust the effective field of view for convolution without reducing the size of the output feature map, in the last few blocks of the backbone network. Thus, Atrous convolution can extract denser features at multiple scales while preserving the spatial resolution, which is significant for semantic segmentation.

For both training and testing images, preprocessing and postprocessing steps were applied. An operator determines slices associated with the top and bottom of the heart, a quick manual step that ensures that images are examined for potential anomalous issues. Heart slices are automatically divided into two equal halves where the lower half was sequenced from bottom-to-middle and the upper half from top-to-middle. Noise reduction was applied. Each $k$ image slice of interest was concatenated with its consecutive two slices and fed to the trained deep network. The output binary mask was applied to the $k$ image slice. As in manual segmentation, we applied noise reduction (3 × 3 × 3 median) to reduce artifacts in these low-dose CT images. We then applied standard fat thresholding [− 190 HU, − 30 HU] to identify EAT within the pericardial sac. The complete testing process is illustrated in Fig. S2.

The ASPP was used on the top of the feature map to capture multi-scale object information by applying four parallel Atrous convolutions with different sampling rates. Batch normalization and image-level features were also incorporated into the ASPP by applying a global average pooling at the last feature map of the backbone and concatenating the corresponding results (contains multi-scale features) with batch normalization^16^. The results were then traversed through a 1 × 1 convolution with 256 filters to obtain the final output. To gradually recover the spatial information and capture more detailed boundary features, a decoder section was added by applying a few 3 × 3 convolutions to refine the output features obtained from the ASPP with an upsampling factor of 4^22^. The complete preprocessing, augmentation, and training are presented in Fig. 2, while the internal structure of DeepLab-v3 plus is illustrated in Fig. S1.

Deep learning experiments were performed using a Windows 10 computer with an AMD Ryzen 7 5800X 3.8 GHz, 32 GB RAM, 1 TB hard disk, and GTX 3090 with 24 GB GPU. We implemented the code using Matlab 2021a. The manual segmentations were implemented on conventional computers using Slicer 3D software, Version 4.11^21^, and the results of manually labeled volumes were saved in DICOM files for easy association with the original CT volumes. For training, we used the Adam method for optimization and Dice as the loss function, as it is immune to the effects of prevalence. To enrich the training process, significant random augmentations were applied. Random rotation (− 5 to 5 degrees), scaling (0.9 to 1.1), and randomized Gaussian blurring with a standard deviation ($\sigma <2$) were applied for data augmentation. We duplicated each input image slice and applied random blurring augmentation to it. Then, with each new training epoch, input images were augmented with a random mixture of rotation and scaling augmentations, creating a wide range of image permutations to enhance the training process. As we typically used 30 epochs for the 50 image volumes, each with an average of 29 image slices, we presented to the network with 1446 input image slices, duplicating them with blur augmentation to 2,892 and up to 86,760 images following randomized augmentation. Using this level of augmentation improves training, especially with a limited dataset^23^. We used a mini-batch strategy with a batch size of 20, while the maximum number of epochs was set to 30 and the initial learning rate was set to 1e-3. Validation was performed at the end of each epoch to evaluate the performance of training and inquire stopping conditions. Training was stopped when changes in Dice reached a tolerance of 0.1e-4 or the maximum number of epochs was reached. We found that training reached an acceptable convergence typically with only 30 epochs.

## Dataset and evaluation methods

This study included 93 non-contrast cardiac CT images, which were obtained from the University Hospital of Cleveland. Four of 93 images were excluded due to abnormality in anatomical structure. For the remaining 89 images, the first and last slices of the thoracic CT volume were manually chosen by analysts to include the heart top and bottom slices, respectively. The axial slice thickness was 2.5 mm and the 2D slice dimensions were 512 × 512 pixels per axial slice, with pixel-spacing ranging from 0.66 to 0.86 mm. A total of 1446 axial slices were included in this study. The dataset was first randomly separated into two sets: training (n = 50) and testing (n = 39). The training set was further divided into two subsets: training subset (n = 40) and validation subset (n = 10). To determine the importance of the processing steps (*HU-attention-window* and *bisect*), we processed the images with and without these modifications.

We evaluated processing using Dice and Intersection Over Union (IOU) scores. The Dice score coefficient was calculated on a slice-by-slice basis for EAT between automated output and the ground truth (manual segmentation) to evaluate the performance of the semantic segmentation. The Dice score ranges from 0 to 1 (0%-100%) with 0 meaning no overlap of segmentation and 1 meaning identical (completely overlapped) segmentation. We evaluated the Dice score using Eq. (1) and reported measures as percentages.

1 $$Dice\left(A,B\right)= \frac{2\times \left|A\cap B\right|}{\left|A\right|+\left|B\right|},$$

where, $A$ and $B$ represent the testing output and the ground truth pixels in a slice (or voxels in a volume), $|A\cap B|$ is the number of overlapping pixels (or voxels) between the predicted EAT segmentation and the ground truth EAT images, and $|A|+|B|$ represents the total number of pixels (or voxels) in both images (or volumes). In our experiment, Dice score coefficients were calculated for both axial 2D slices and the whole 3D volume. We also calculated the IOU score, also known as the Jaccard Index. Similar to the Dice score, a 0 value indicates no overlapping segmentation and 1 represents identical segmentation, as follows:

2 $$IOU\left(A,B\right)= \frac{\left|A\cap B\right|}{\left|A\right|+\left|B\right|-\left|A\cap B\right|} .$$

In addition, to help identify any algorithm issues, we compared the automated EAT volumes to those from analyst manual segmentations. Scatter and Bland–Altman plots were created across volumes and image slices to evaluate the agreement between the predicted results and the manual ground truth. The correlation coefficient (R) and its corresponding p-value were calculated to assess the scatter plots.

## Results

**Figure 3:**
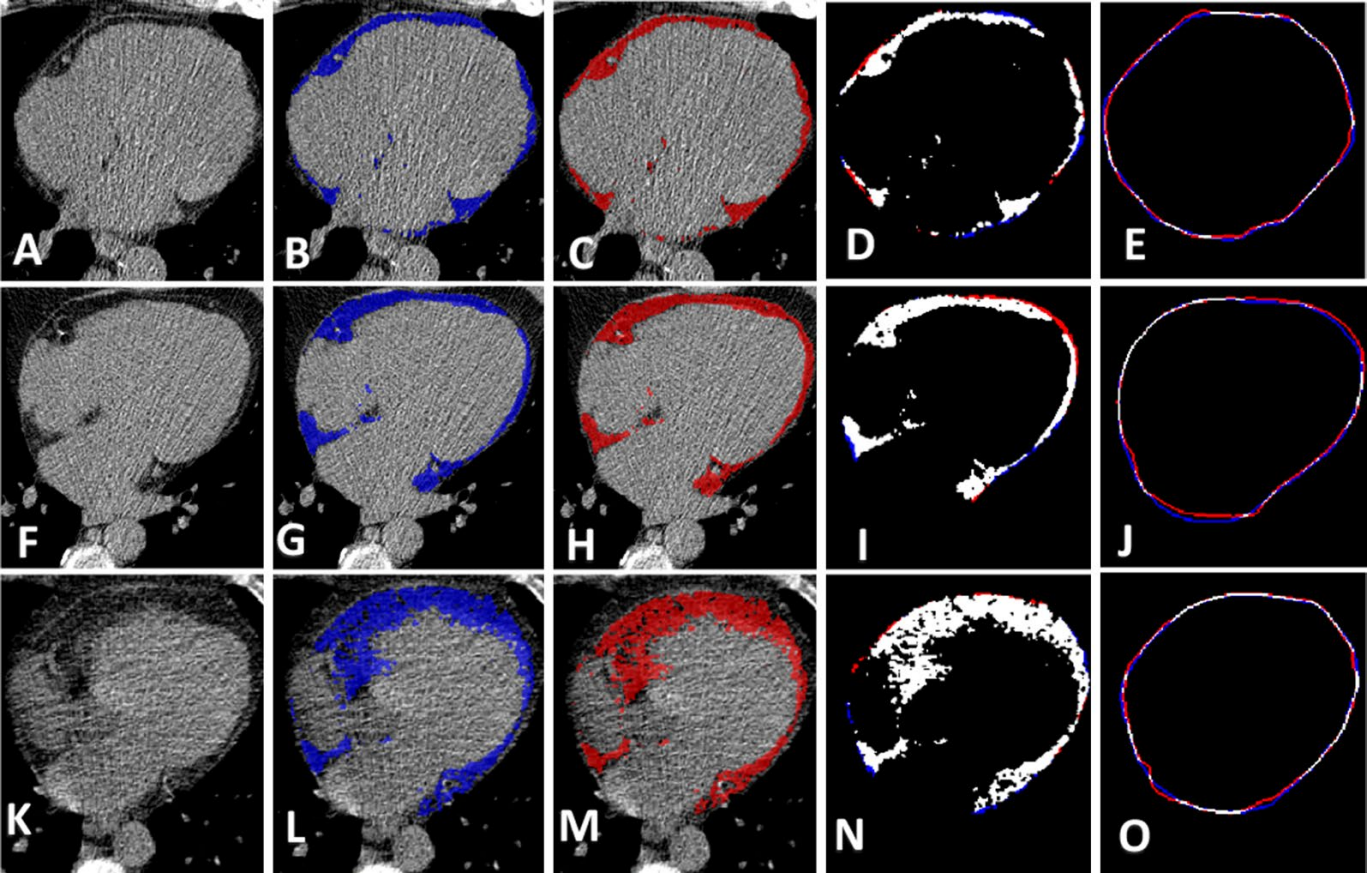
Automated segmentation of epicardial adipose tissue (EAT). Axial non-contrast CT images (**A,F,K**), manual segmentation in blue (**B,G,L**), and automated segmentation (using DeepFat with the bisect method) in red (**C,H,M**). Combined manual and automated EAT segmentation is shown in (**D,I,N**), where red represents manual, blue represents automatic, and white is the overlapping area. Pericardial sac contours using the same color scheme are shown in (**E,J,O**). The total subject EAT Dice score is 86.8%, 92.3%, and 92.4%, in the rows with low (**A–E**), intermediate (**F–J**), and high fat (**K–O**). Errors tend to be at the edges of the pericardial sac.

DeepFat showed excellent segmentation of the pericardial sac and EAT. In Fig. 3, we compare DeepFat EAT segmentations to the manually obtained gold standard image results in three held-out test volumes. There was good agreement and only small deviations in the marking of the pericardial sac (Fig. 3E,J,O). Dice scores for EAT for these images were 86.8%, 92.3%, and 92.4% (Fig. 3A,F,K, respectively).

**Figure 4:**
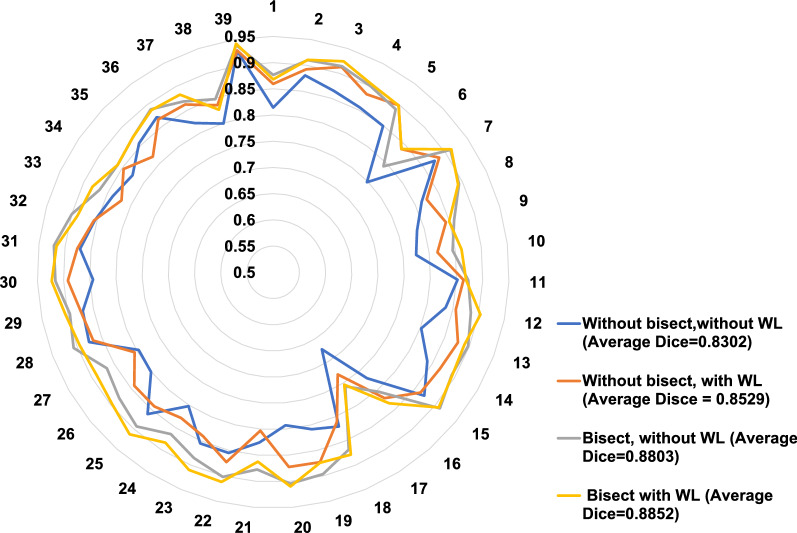
Comparison of Dice scores for DeepFat with and without *HU-attention-window* and *bisect.* Plot shows Dice scores for the 39 images in the held-out (testing) set. Dice was calculated against the manual ground truth. Average Dice scores for without *bisect*, without *HU-attention-window* (WL); without *bisect*, with *HU-attention-window*; with *bisect*, without *HU-attention-window*; and with *bisect*, with *HU-attention-window*, are 83.0% ± 4.5, 85.3% ± 3.6, 88.0% ± 3.5, and 88.5% ± 3.4, respectively. See text for other analysis details.

We evaluated the contributions of our algorithm choices (e.g., *HU-attention-window* and *bisect*) in a radar graph (Fig. 4). Comparing the results with HU-attention-window, volume Dice scores were much improved with *bisect* compared to without *bisect.* The addition of bisect resulted in the best Dice score in 95% of test volumes. Likewise, *HU-attention-window* improved results in the presence of *bisect*, with better Dice scores in 64% of tested volumes. Using both *HU-attention-window* and *bisect* was significantly advantageous, providing an improved Dice score in 100% of tested volumes, indicating the usefulness of these algorithm choices. Average Dice scores also showed the value of including both *HU-attention-window* and *bisect* (see Fig. 4 legend). Data augmentation was also found to be important, especially with image blurring, giving 1.77% absolute improvement in Dice.

When we investigated the use of different deep learning networks (Table S1), we found that DeepLab-v3 Plus outperformed the three other networks tested. Improvements were surprisingly substantial, with absolute improvements in Dice ranging from 25 to 2% and IOU from 33 to 2%, depending on the network.

**Figure 5:**
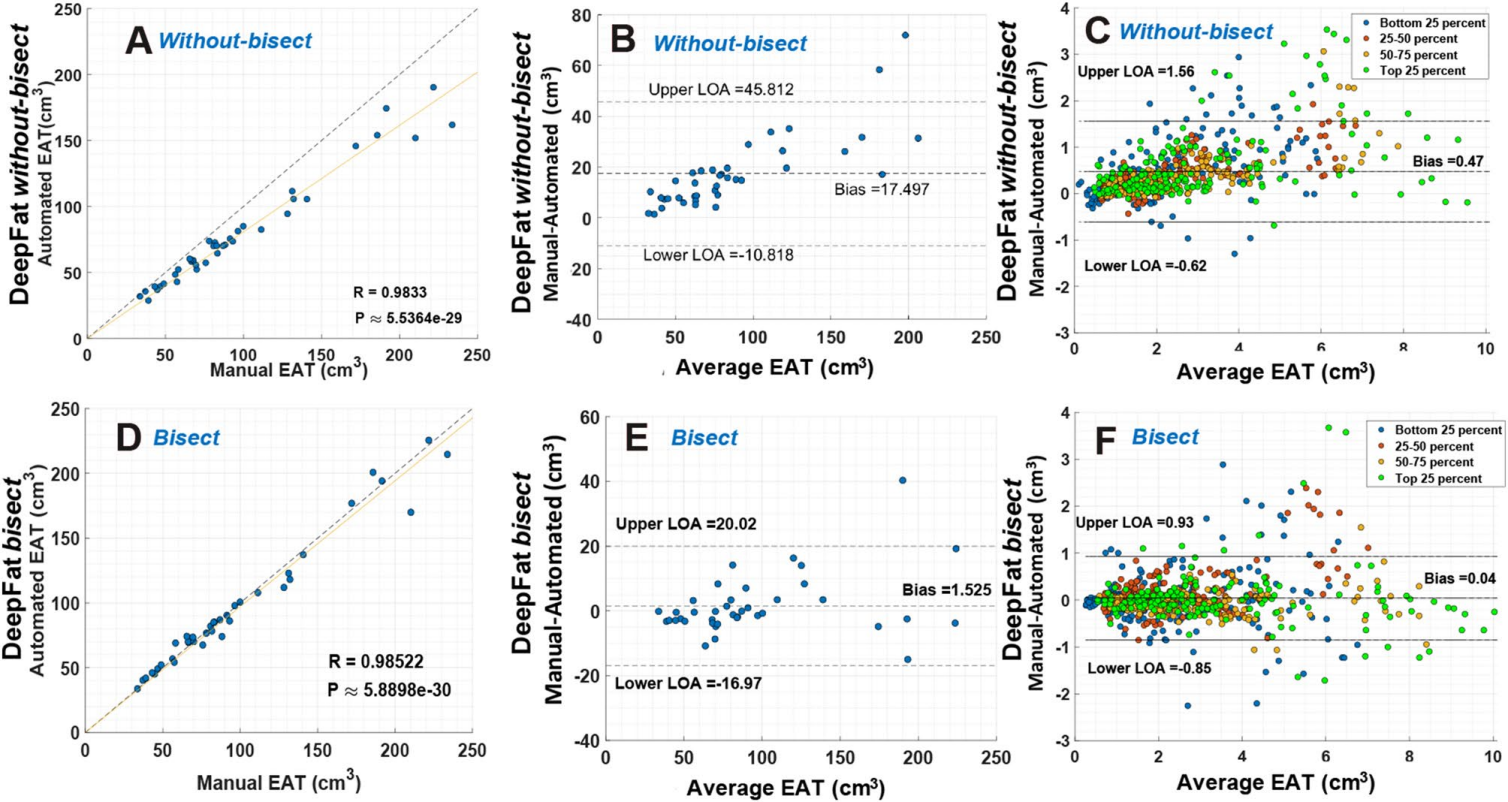
Impact of *bisect* on automated EAT volume analysis with DeepFat. With *bisect*, data points are clustered near the line of identity (**D**), giving much better results than without *bisect* (**A**). To evaluate the data, we computed R values and slopes from a fit of y = mx, which gives slopes (R) of 0.807 (0.9833) and 0.971 (0.9852), for (**A,D**), respectively, again showing the value of the *bisect* modification. Comparing Bland–Altman plots (**B,E**), the bias and spread (limits of agreement or LOA corresponding to1.96 × standard deviation) are both reduced for *bisect* compared to *without-bisect*. Bias with *bisect* (**E**) is 1.5 cm^3^, on the order of only 1% of measured values. In the Bland–Altman plot (**E**), the single largest outlier for DeepFat at + 40 cm^3^ has an unusual automatic segmentation, which is easily identified and corrected. (**C,F**) Show results for image slice volumes, with slices color coded as to location in the heart. In general, image slices at the top and bottom of the heart have the most errors; this is improved with the *bisect* modification, resulting in better than 90% reduction in bias to only 0.04 cm^3^.

Good segmentations translated to good EAT volumes with DeepFat. With and without *bisect,* we compared automatically obtained DeepFat total EAT volumes to manually obtained results (Fig. 5). The deep network with *bisect* gave superior total EAT volume estimation compared to the deep network without *bisect*, as shown in both scatter and Bland–Altman plots (first two columns). R, slope, bias, and spread values all improved with bisect (see Fig. 5 legend). It is understood that R is a weak assessment tool for the quality of the measurement, as it does not indicate the quality of the y = mx fit. Assessments per slice allowed us to analyze and optimize the algorithm (Fig. 5C,F). The slices of each test image were categorized into four equal regions based on their location in the total heart slice sequence and regions were color-coded. Without using *bisect* method, image slices at the top and bottom of the heart tended to have the most error. This was reduced with the inclusion of *bisect*, due to the ability of *bisect* to capture the heart shape near the top and bottom of the heart. A plot such as those shown in Fig. 5C,F helped us diagnose and optimize our DeepFat algorithm, leading to the creation of the *bisect* modification, where slope, bias, and spread were greatly improved (also presented in scatter plot shown in Fig. S3). As the HU value of fat has been implicated as an indicator of fat inflammation, the mean HU value of fat was analyzed. DeepFat gave whole heart mean epicardial fat HU values very close to those obtained manually (Fig. S4A), with R = 0.998 and a bias of 0.35 HU. A slice-based plot gave similarly excellent results (Fig. S4B). As the fat range is wide (− 190, − 30), a bias of less than one HU unit (0.35 HU) is excellent. It is also less than the standard deviation of 1.1 HU. These observations indicate excellent agreement with the manual result.

**Figure 6:**
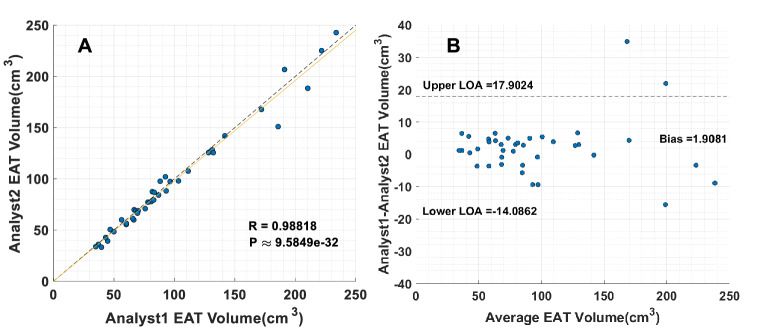
Comparison of total EAT volumes manually analyzed by two different analysts. Good agreement is observed between analyst1 and analyst2 in both the scatter plot and Bland–Altman plot. Bias is small (1.9 cm^3^), only 1–2% of the measured volumes. Nevertheless, there are substantive differences for some images, shown as outliers. For example, the largest negative outlier in the Bland–Altman plot has a difference of approximately − 35 cm^3^, or a 20% percent difference. In such volumes, the pericardial sac is not clearly identified, likely due to motion or noise in larger patients.

**Figure 7:**
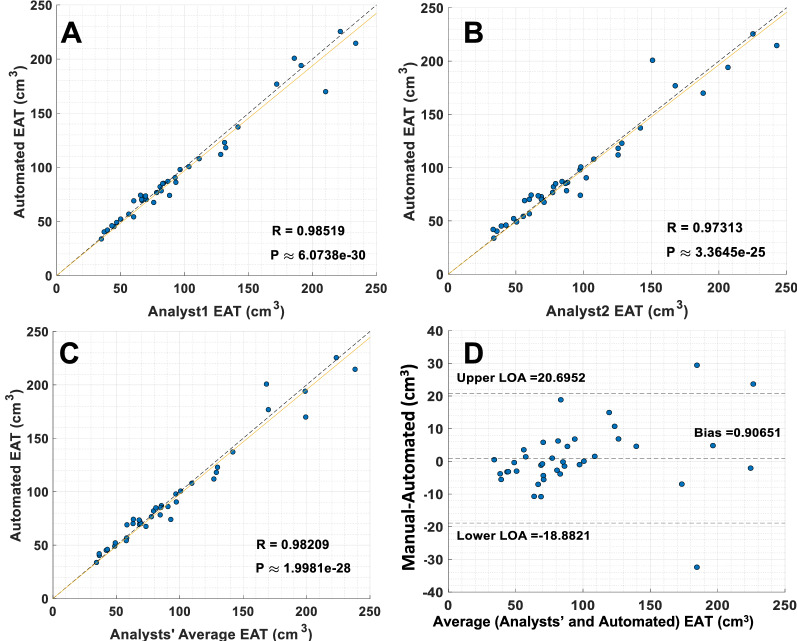
Comparison of total EAT volumes analyzed by DeepFat and the two analysts. A slightly higher correlation is found with Analyst1 (**A**) than with Analyst2 (**B**), indicated by R = 0.9852 and R = 0.9731, respectively. (**C,D**) Compare DeepFat to volumes averaged for Analyst1 and Analyst2, giving R values only slightly inferior to that for Analyst1. Scatter plot of DeepFat volumes against the average of Analyst1 and Analyst 2 (**C**) is visually comparable to that between Analyst1 and Analyst2 in Fig. 6, indicating that DeepFat performs well compared to analysts. Bland–Altman plot (**D**) compares favorably to that for Analyst1 versus Analyst2 in Fig. 6. Average-DeepFat shows a 50% reduction in bias compared to Analyst2–Analyst1. The spread with average-DeepFat is only a little larger (20%) than that for Analyst2-Analyst1. Note that all results in this figure and in Fig. 6 come from the same held out (testing) set of images.

We analyzed the variation between analysts (inter-reader variability) (Fig. 6) and compared it to the variation between analysts and the DeepFat automated method (Fig. 7). The 50 training CT images were split into groups of 25, 12, and 13 images that were manually analyzed by analyst1, analyst2, and analyst3, respectively. In the inter-reader variability study, we compared the manual segmentation of analyst1 versus analyst2 over the 39 held-out testing set. Scatter and Bland–Altman plots between the two analysts are shown in Fig. 6. There was reasonable agreement (R = 0.9882, p < 0.001) between the two analysts; however, the standard-deviation/bias from the Bland–Altman plot (8.16 cm^3^/1.91 cm^3^) indicated variability. The largest outlier showed a difference of + 35 cm^3^ out of 169 cm^3^, a 20% difference. Inspection of this image showed that in some slices, expert analysts disagreed on the placement of the pericardial sac. Figure 7 shows scatter plots comparing the segmentation from the same two analysts against DeepFat for the same 39 testing set of images. There was good agreement with both analyst1 and analyst2 (R = 0.9852 and R = 0.9731, respectively). Dice scores were 88.53% and 87.24% against analyst1 and analyst2, respectively. Interestingly, there was a slightly better agreement with analyst1 than analyst2, probably because analyst1 had labeled more volumes in the training set than analyst2. In both Figs. 6 and 7, Bland–Altman plots show increased differences at higher volumes, probably indicating that an error in the placement of the pericardial sac can result in a larger volume difference. As there was relatively little difference between analyst1 and analyst2, we averaged their volumes for further analysis of DeepFat (Fig. 7C,D). The scatter plot of DeepFat volumes against the average of analyst1 and analyst2 (Fig. 7C) was visually comparable to that between analyst1 and analyst2 in Fig. 6A, indicating that DeepFat performed well as compared to the analysts. Similarly, we found that the Bland–Altman plots (Figs. 6A, 7D) compared favorably, with the bias reduced by > 50% with DeepFat.

## Discussion

Our novel fully-automated DeepFat method for automated analysis of EAT in non-contrast CT images showed excellent results, in terms of Dice score and measured EAT volumes. Automatically obtained volumes compared favorably to manually obtained values with a percent difference of only 0.91% $\pm$ 10.1. When the gold standard is manual analysis, an exacting criterion for automated analysis is that it falls within the uncertainty of analysts. DeepFat met this criterion. When we plotted EAT volumes for analyst1 versus analyst2, data clustered near the idealized line (Fig. 6, R = 98.8). We saw similar visual results when DeepFat volumes were plotted as a function of the average of the two analysts (Fig. 7C,  R = 98.2), suggesting that agreement of DeepFat with the analysts is about as good as the agreement of one analyst with another. The bias of DeepFat values was very small (0.9 cm^3^), considering that many fat volumes exceed 100 cm^3^. The percent difference for DeepFat (0.91% $\pm$ 10.1) compared favorably to the percent difference between analysts for EAT (1.91% $\pm$ 8.1). Although paired t-test indicated that the mean difference did not meet the requirement for insignificant difference from zero, the p-value for DeepFat/mean-of-analysts was p = 0.29, comparable to that for analyst1/analyst2 (p = 0.07), again indicating that DeepFat performed well compared to the analysts. Omitting the single outlier (image with an average-automated difference of − 32.4 cm^3^), gave even better results, with paired t-test p values of 0.10 and 0.15 for DeepFat/mean-of-analysts and analyst1/analyst2, respectively. Altogether, these findings imply that the automated DeepFat algorithm performs as well as the analysts for measuring EAT.

We note some important aspects of the DeepFat algorithm revealed by our study. First, deep learning segmentation of the region inside the pericardial sac was superior to methods that try to identify the thin contour of the sac directly. Regional segmentation allowed us to use the Dice loss function and avoid the large class imbalance that we would see with contour segmentation. Second, we determined that it is important to use *HU-attention-window*. Otherwise, small contrasts will be lost in creating 8-bit data in the DeepLab-v3 plus implementation used by us or in numerical optimization of weights. We found this to be an important step in EAT deep learning segmentation. Only one other group (Commandeur et al.^5^) includes this as a pre-processing step. Third, the *bisect* method greatly improved segmentations at the top and bottom of the heart. Essentially, using the look ahead slab-of-slices allowed the deep learning algorithm to learn the curvature of the sac at the top and bottom of the heart, imitating the way analysts examine adjacent slices when segmenting the sac. Adding the *bisect* step improved the Dice score from 85.3 to 88.5% (Fig. 4). Fourth, augmentation played a key role, as it enriched the deep learning with variations of cases to train the network. In particular, we found that it was important to add the image blurring augmentation. Finally, DeepLab-v3 plus was found to be superior to other networks (Table S1).

The slice-based plots, which to our knowledge had not been investigated previously, provided a detailed per-slice segmentation evaluation. Since the deep network tries to learn the EAT per slice, this study revealed the regions where the network suffers from in-quartile grouped slices. Detailed slice-by-slice plots made it possible to distinguish the deep learning difficulties in segmenting the upper and lower slices in a *without bisect* method, which underscores the need for our *bisect* method (Fig. 5C,F, Fig. S3).

We compared our results to those in four recent publications (Table 1). DeepFat with *bisect* compared favorably to all methods despite differences in algorithms, cohorts, and imaging methods. DeepFat achieved the best R-value among all methods. It gave the best Dice score in publications using non-contrast CT. In comparison with CTA studies, DeepFat results were only slightly less (0.18% absolute Dice) than high resolution and high contrast CTA (e.g., He et al.^9^). This difference is likely insignificant given statistical variations. Note that CTA has a thinner slice (0.5 mm thickness), thus producing 5 times the total number of slices than non-contrast CT images 2.5 mm thickness, and CTA uses a contrast agent that further improves the detection of fat. It is surprising that our method on CT calcium score images performs equally well.

**Table 1:** Comparison of DeepFat results to results reported in previous studies.

Image type	Study	Cohort population	Deep technique	Average dice score (%)	Correlation coefficient R (%)
CTA	He^18^	40	3D deep attention U-Net	85	–
	He^15^	200	3D deep attention U-Net	**88.7**	**94.9**
CT	Commanduer^20^	250	2 CNNs	82.3	92.4
	Commanduer^13^	614	2 CNNs	87.3	97.4
	DeepFat (without *bisect*)	89	DeepLab-v3 plus	85.29 $\pm$ 3.59	98.3
	**DeepFat (with *bisect*)**	89	DeepLab-v3 plus	**88.52** $\pm$ **3.35**	**98.5**

*CNN* convolutional neural network, *CT* thick-slice non-contrast CT, *CTA* thin-slice with contrast agent.

Significance values are given in bold.

In conclusion, our automated DeepFat EAT segmentation method with *HU-attention-window* and *bisect* improvements outperformed methods reported in recent studies to quantify EAT in CT images. The method appears to be appropriate for use in substantive population studies. Nevertheless, we plan to perform a manual review of the automated results to further investigate errors that are readily identified (e.g., the outlier described above). As we gather more training data, possibly from manual corrections of automated segmentations, we anticipate even better performance with DeepFat.

## Supplementary Information

Supplementary Information.
