# Supplementary material for: Deep learning segmentation and quantification method for assessing epicardial adipose tissue in CT calcium score scans
Source: Sci Rep. 2022 Feb 10;12:2276. doi: 10.1038/s41598-022-06351-z (PMC8831577; doi:10.1038/s41598-022-06351-z)
Supplement: Supplementary file 1 — Supplementary Information. [file 41598_2022_6351_MOESM1_ESM.pdf]

Supplemental Tables and Figures

We investigated the use of different deep learning techniques with DeepFat, either with or without *bisect*, as shown in Table S1. The study included training on the same 50 CT scans and testing on the same 39 CT scans using the DeepFat method, while changing the internal deep network block (see Figure 1 step 4). We compared U-Net (with CNN as internal blocks), Fully Convolutional Network (FCN, with VGG-16 internal blocks), SegNet (with VGG-16 internal blocks), and our DeepLab-v3 Plus (using ResNet-18). Our method provided the highest average Dice score of 88.52%± 3.35 and the highest average IOU score of 79.38%±4.71, with a small average EAT volume error of 0.5%±8.1. Overall, results from *bisect*-based slice organization were better than those from *without bisect,* across all three deep networks. This analysis emphasized the advantage of the DeepFat method associated with the suggested DeepLab-v3 Plus deep network.

Figure S1 shows the internal structure of the DeepLab-v3 Plus used for segmentation of the region within the pericardial sac. In the training phase, the input slab with $k, k+1,$ and $k+2$ slices is provided with the binary labeled, *k*, output image (region interior to sac contour). The encoder, ASPP, and decoder parts of the semantic segmentation are presented. In the testing phase, the trained deep network predicts the enclosed labeled of sac region output.

Figure S2 demonstrates the full structure of the automated EAT segmentation testing phase process with preprocessing steps. Similar to the training phase, we divide the heart slices into two halves (50% of total slices upper and 50% lower half) where the lower half is sequenced from bottom-to-middle and the upper half sequenced top-to-middle, thereby keeping an increasing curvature of the sac (bisect method). The trained DeepLab-v3 plus network is tested with each of the three sequenced patches generating a single binary corresponding mask slice.

Figure S3 compares the automated DeepFat (with and without *bisect*) versus the manual EAT volumes from each CT slice. The regions are presented in color: blue for slices in the bottom 25%, brown for slices between 25%-50%, orange for slices between 50%-75%, and bright green for slices in the top 25%. A scatter plot of results from the without-bisect method shows a correlation of R=0.9584 (p<0.001) while results from the bisect method show correlation of R=0.9662 (p<0.001).

Figure S4 provides analytical Bland-Altman plots for the proposed (DeepFat with bisect) effects on mean HU values. A patient-based plot (A) shows an almost identical correlation (R=0.998) between DeepFat and manual mean HU values with a negligible bias of (0.35 HU). More intuitively, a slice-based Bland-Altman plot with regional labeled-grouped slices shows less but great correlation (R=0.992) with the same small mean HU bias of (0.35 HU) between automated and manual segmentation results. These negligible results prove that the proposed method provides almost identical HU mean values and did not change their distribution, as the fat range is wide (-190, -30) compared to a bias of less than one HU unit (0.35) and mean HU value difference less than 1.1 HU.

TABLE S1

DEEPFAT RESULTS OF DIFFERENT DEEP SEGMENTATION NETWORKS USING THE SAME TRAINING/TESTING COHORT WITH/WITHOUT BISECT METHODS

Deep Segmentation Network	Slice organizing method	Average Dice Score (%)	Average IOU Score (%)	Average EAT volume error (%)
U-Net (CNN)	*without bisect*	62.81±9.47	46.43±9.63	-51.24±10.3
U-Net (CNN)	*with bisect*	63.24±9.47	46.89±9.76	-52.78±9.8
FCN (VGG-16)	*without bisect*	77.38±7.31	63.64±9.19	25.47±31.91
FCN (VGG-16)	*with bisect*	79.43±5.71	66.22±7.49	-7.79±14.99
SegNet (VGG-16)	*without bisect*	85.23±4.22	74.50±6.25	18.82±15.3
SegNet (VGG-16)	*with bisect*	86.63±4.07	76.62±6.01	**-0.18±8.9**
SegNet (VGG-19)	*without bisect*	85.92±3.36	75.47±5.04	0.36±10.7
SegNet (VGG-19)	*with bisect*	86.98±3.69	77.14±5.61	-3.45±9.6
DeepLab-v3 Plus (ResNet-18)	*without bisect*	85.29± 3.59	74.52±5.35	21.04±10.1
**DeepLab-v3 Plus (ResNet-18)**	*with bisect*	**88.52±3.35**	**79.38±4.71**	0.5±8.1

CNN, Convolutional Neural Network; EAT, epicardial adipose tissue; IOU, Intersection Over Union; FCN, Fully Convolutional Network.

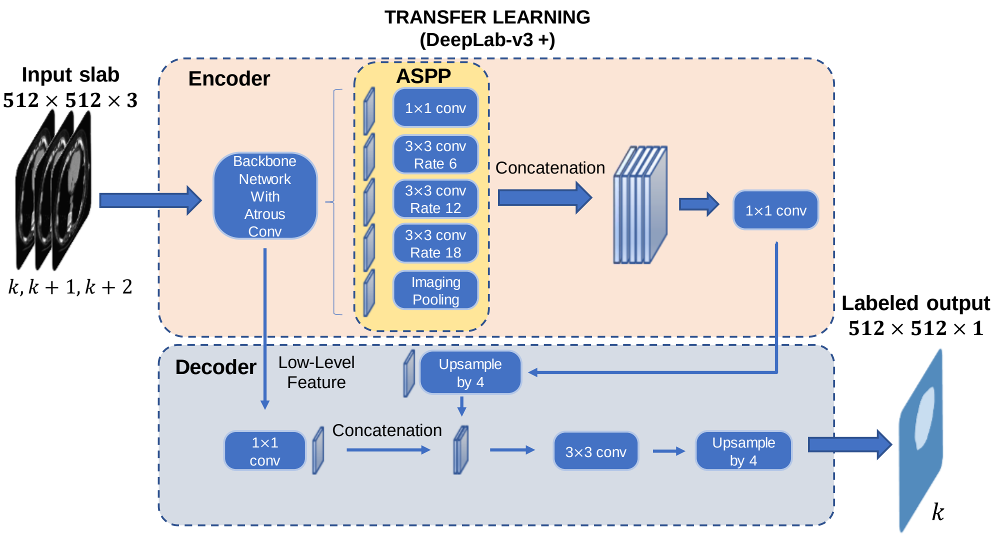

Figure S1. The internal structure of the DeepLab-v3 Plus used for segmentation of the region within the pericardial sac. This image expands on item 4 in Fig. 2. During training, the input slab of three image slices is provided with the binary labeled output image (the region interior to sac contour). The encoder, ASPP, and decoder parts of the semantic segmentation are shown (see text for details). In the testing phase, the trained deep network predicts the untrained k slice labeled output.

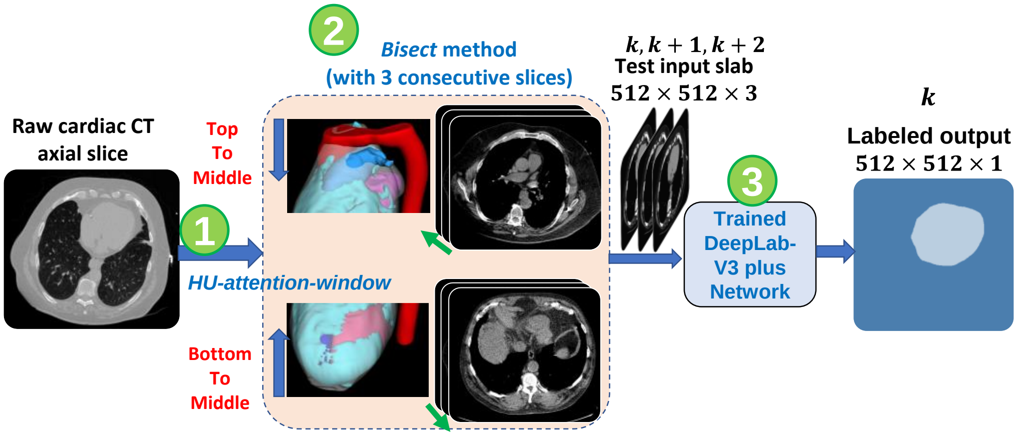

Figure S2. The full structure of the automated EAT segmentation testing phase process with preprocessing steps. Like training phase, an HU-attention-window/level of 40 HU/350 HU is shown in (1). A look ahead slab-of-slices with increasing size is presented to the network with the slice of interest and two up-coming slices on the increasing side, as in (2). As in the training phase, we divide the heart slices into two halves (50% of total slices upper and 50% lower half) where the lower half is sequenced from bottom-to-middle and the upper half sequenced top-to-middle, thereby keeping an increasing curvature of the sac (bisect method), as in (2). Finally, the trained DeepLab-v3 plus network is tested with each of the three sequenced patches generating a single binary corresponding mask slice as in (3).

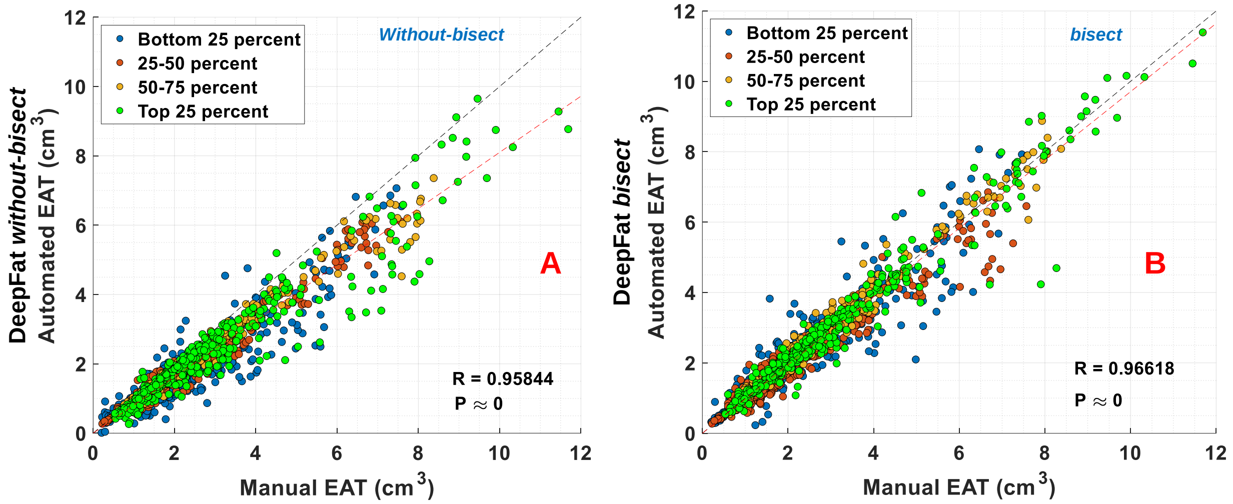

Figure S3. Comparison of automated DeepFat (*without-bisect* and *bisect*) versus manually segmented EAT volumes from each CT slice. Colors represent axial location in the heart from the bottom 25% (towards the apex) to the top 25%. (towards the base). Compared to *without-bisect* (A), DeepFat with *bisect* (B) gives a tighter distribution that lies closer to the ideal line of slope 1.0. Particularly, the *without-bisect* graph shows slices in the top 25% (bright green) are spread and tend to undershoot the ideal line. In both cases, measurements in the middle of the heart (brown and orange) tend to cluster together. Analyzing only slices from the top 25% (bright green) and the bottom 25% (blue), fitting DeepFat with *bisect* data to the equation y=mx gave a slope of 0.971, very near the ideal value of 1.0 and much better than the value of 0.81 obtained for the DeepFat *without-bisect* method.

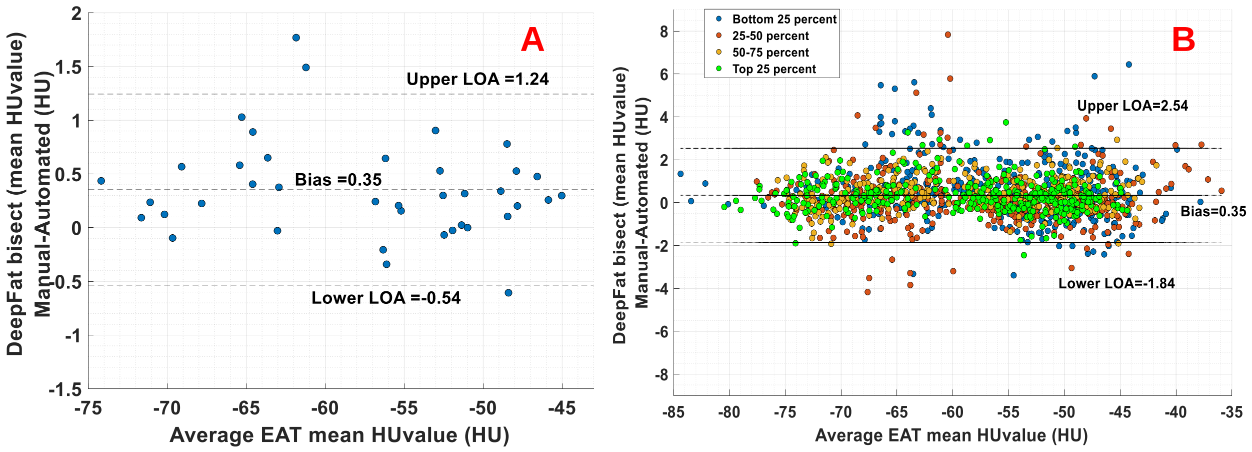

Figure S4. Impact of the proposed automated method (DeepFat with bisect) on EAT mean HU values. For per patient Bland-Altman plot, the DeepFat method shows great correlation (R=0.998) with manual mean HU values with negligible bias of (0.35 HU) (A). Similarly, a slice-base Bland-Altman plot with regional labeled-grouped slices (B) shows great correlation (R=0.992) with small mean HU bias of (0.35 HU) between automated and manual segmentation results. The bias and spread (limits of agreement or LOA corresponding to 1.96x standard deviation) are within significantly small HU ranges compared to the large Fat window of (-190, -30).
